# Reduced inhibitory action of a GABA_B _receptor agonist on [^3^H]-dopamine release from rat ventral tegmental area *in vitro *after chronic nicotine administration

**DOI:** 10.1186/1471-2210-4-24

**Published:** 2004-10-20

**Authors:** Diana Amantea, Norman G Bowery

**Affiliations:** 1 Department of Pharmacology, Medical School, University of Birmingham, Edgbaston, Birmingham B15 2TT, UK; 2 Department of Pharmacobiology, University of Calabria, 87036 Rende, Cosenza, Italy

## Abstract

**Background:**

The activation of GABA_B _receptors in the ventral tegmental area (VTA) has been suggested to attenuate the rewarding properties of psychostimulants, including nicotine. However, the neurochemical mechanism that underlie this effect remains unknown. Since GABA_B _receptors modulate the release of several neurotransmitters in the mammalian brain, we have characterised the effect of the GABA_B _receptor agonist baclofen on the release of [^3^H]-dopamine ([^3^H]-DA) from VTA slices of naïve rats and of rats pre-treated with nicotine.

**Results:**

In naïve rats, baclofen concentration-dependently inhibited the electrically evoked release of [^3^H]-DA from the isolated VTA (EC_50 _= 0.103 μM, 95% CI = 0.043–0.249), without affecting the basal [^3^H]-monoamine overflow. This effect was mediated by activation of GABA_B _receptors as it was blocked by the selective receptor antagonist CGP55845A. Chronic administration of nicotine (0.4 mg kg^-1^, s.c., for 14 days) affected neither the basal nor the electrically evoked release of [^3^H]-DA from VTA slices. However, the inhibitory effect of baclofen (10 μM) on the stimulated [^3^H]-monoamine overflow was abolished in rats pre-treated with nicotine as compared to saline-injected controls.

**Conclusions:**

Our results demonstrate that GABA_B _receptor activation reduces the release of DA from the rat VTA. In addition, a reduced sensitivity of VTA GABA_B _receptors appears to develop after chronic exposure to nicotine. The resulting disinhibition of VTA DA neurones might therefore contribute to the sensitised dopaminergic responses observed in the rat mesocorticolimbic system following repeated administration of nicotine.

## Background

The ventral tegmental area (VTA) represents the site of origin of the mesocorticolimbic dopaminergic pathway that has been implicated in mediating the reinforcing properties of drugs of abuse, including nicotine [1-3].

The majority of cells within the ventral tegmental area consist of dopaminergic, tyrosine-hydroxylase containing neurones, which send axon projections to forebrain structures such as the nucleus accumbens and the prefrontal cortex. The non-tyrosine hydroxylase containing neurones are mainly GABAergic and function either as local interneurones to modulate the activity of the principal dopaminergic cells or as projection neurones providing inhibitory input to the cortex and the nucleus accumbens [4-8].

In addition to classical release from the axon terminals located in the forebrain, midbrain dopaminergic neurones release dopamine (DA) from their soma and dendrites [9-12]. The somatodendritic release of DA provides a primary modulation of dopamine cell function. Activation of D_2 _autoreceptors inhibits excitability and firing rate of VTA dopaminergic neurones [13,14] and decreases the release of dopamine from their axon terminals in the forebrain [15,16]. Furthermore, somatodendritic dopamine can indirectly modulate the activity of midbrain dopamine cells by acting on D_1 _receptors, which are found on GABA- and excitatory amino acids-containing terminals in the VTA [17-20].

Besides the short-loop feedback inhibition exerted by dopamine, the activity of VTA dopaminergic neurones is strongly modulated by glutamatergic and GABAergic inputs. Activation of NMDA receptors by excitatory afferents arising from the medial prefrontal cortex [21,22] induces burst firing in VTA DA neurones [23-25], which is associated with increased dopamine release from the nerve terminals in the nucleus accumbens [26,27]. The VTA also receives an extensive inhibitory influence arising from GABA interneurones and descending projections from the basal forebrain, innervating GABA_A _and GABA_B _receptors, respectively [4]. Activation of GABA_B _receptors has been reported to inhibit the spontaneous pacemaker-like activity of VTA DA neurones in slice preparations [4,28,29] and to decrease the firing rate and burst firing of these cells *in vivo *[30,31].

In addition, *in vivo *microdialysis studies have demonstrated that intra-VTA administration of the GABA_B _receptor agonist baclofen decreases extracellular dopamine levels in both the somatodendritic [32,33] and the axon-terminal regions of the mesocorticolimbic system [15,16]. The GABA_B _receptor-mediated inhibition of the activity of the mesocorticolimbic neurones might explain the effectiveness of baclofen to suppress nicotine self-administration when microinjected into the VTA [34]. In fact, the rewarding properties of nicotine have been ascribed to its ability to stimulate VTA dopamine neurones that project to the nucleus accumbens [2,35]. Acute administration of nicotine to drug-naïve rats increases extracellular levels of dopamine in the nucleus accumbens shell, while repeated exposure to the drug results in sensitisation of its effect on dopamine overflow in the nucleus accumbens core [36]. Sensitisation of the mesoaccumbens dopamine response to nicotine appears to be closely related to the dependence-liability of the drug and has been suggested to reflect an altered control of DA release, including reduced inhibitory influence by DA autoreceptors, and co-stimulation of NMDA receptors [37-39].

Since GABA_B _receptors have a prominent role in regulating the activity of VTA DA neurones and they appear to be involved in the modulation of nicotine reinforcing properties, we have characterised the effect GABA_B _receptor stimulation on the somatodendritic release of [^3^H]-DA from VTA slices of naïve rats and used this model to determine whether chronic exposure to nicotine results in altered GABA_B _receptor-mediated modulation of VTA DA cells.

## Results

### Tetrodotoxin and calcium dependence of the stimulated [^3^H]-DA release from VTA slices

**Figure 2:**
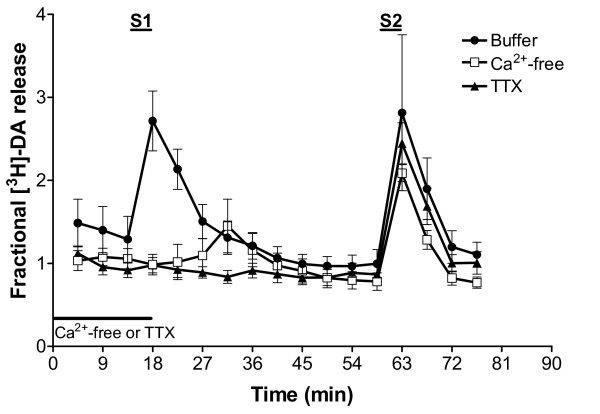
Calcium-dependence and tetrodotoxin (TTX) sensitivity of the electrically evoked release of [^3^H]-DA from ventral tegmental area slices. Electrical stimulation (20 mA, 2 Hz, 4 min) occurred 14 min (S1) and 59 min (S2) after the beginning of sample collection. Slices were perfused with buffer alone (control), calcium-free medium containing 1 mM EGTA (Ca^2+^-free), or buffer containing 1 μM TTX for 15 min before and during S1 (black bar). After 18 min from the beginning of sample collection, all samples were superfused with normal buffer. Values represent mean ± s.e.mean (*n *= 3 rats).

The influence of tetrodotoxin (TTX) and calcium on the electrically evoked release of [^3^H]-DA from ventral tegmental slices of naïve rats was evaluated in order to determine its physiological significance under the experimental conditions used here. Removal of calcium with the inclusion of EGTA (1 mM), or addition of TTX (1 μM) to the superfusion buffer abolished the stimulated release of [^3^H]-DA from the tissue, without significantly affecting the spontaneous monoamine overflow. Recovery of the release occurred during the second stimulation (S2), after removal of TTX or replacement of calcium to the superfusion buffer (Figure 2).

### Effect of GABA_B _receptor activation on VTA [^3^H]-DA release

**Figure 3:**
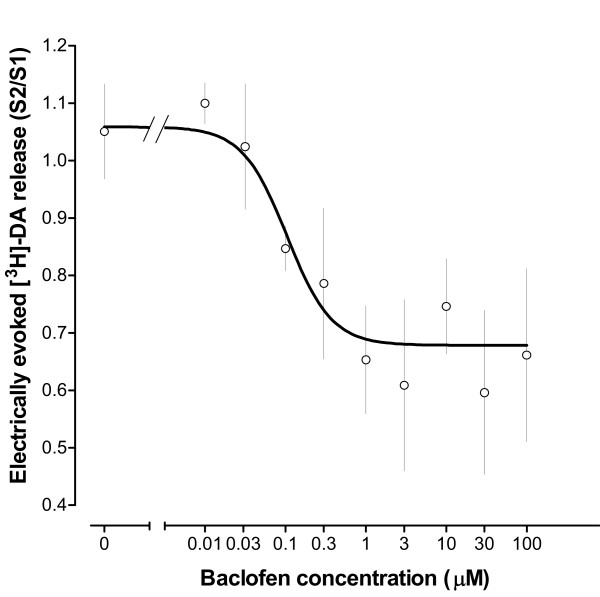
Baclofen-mediated inhibition of the electrically evoked [^3^H]-DA release from ventral tegmental area slices of naïve rats (EC_50 _= 0.103 μM, 95% CI = 0.043–0.249). Baclofen (0.01–100 μM) was added to the superfusion buffer 36 min before and during S2. Values represent mean ± s.e.mean (*n *= 4–7 rats).

Baclofen (0.1–100 μM), added to the superfusion buffer 36 min before the second stimulation, dose-dependently reduced the electrically evoked [^3^H]-DA release from VTA slices (EC_50 _= 0.103 μM, 95% CI = 0.043–0.249, *n *= 4–7 rats, Figure 3), without having any significant effect on the basal monoamine overflow (data not shown).

**Figure 4:**
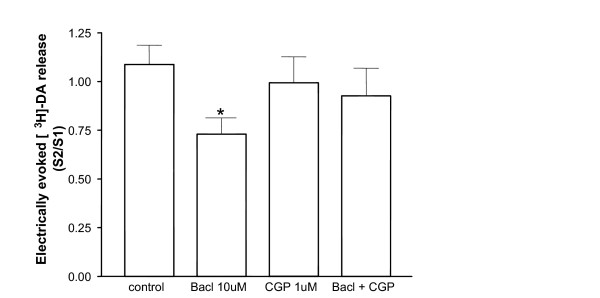
Reversal of baclofen-mediated inhibition of the evoked [^3^H]-DA release from VTA slices by the GABA_B _antagonist CGP55845A. Baclofen (Bacl, 10 μM) and/or CGP55845A (CGP, 1 μM) were added to the superfusion buffer 36 min before and during S2. Drug effects were assessed by comparing the ratio S2/S1 in the presence and absence of the drug, respectively. Values represent mean ± s.e.mean (*n *= 6–10 rats). *p < 0.05 vs control (one-way ANOVA, Dunnett's post hoc test).

The inhibitory effect of baclofen (10 μM) on the release of [^3^H]-DA was abolished when the selective GABA_B _receptor antagonist CGP55845A (1 μM), which had no effect on its own, was added to the superfusion buffer concomitantly with baclofen (Figure 4). Release of dopamine, expressed as S2/S1 ratio, was 1.09 ± 0.10 for control slices perfused with buffer alone; 0.73 ± 0.08 for baclofen added before S2 (P < 0.05 vs control, one-way ANOVA, Dunnet's post hoc test, *n *= 6–10 rats); 0.99 ± 0.13 for CGP55845A alone; and 0.93 ± 0.14 for slices perfused with both baclofen and CGP55845A.

### Effect of nicotine pre-treatment on the release of [^3^H]-DA from VTA

**Figure 5:**
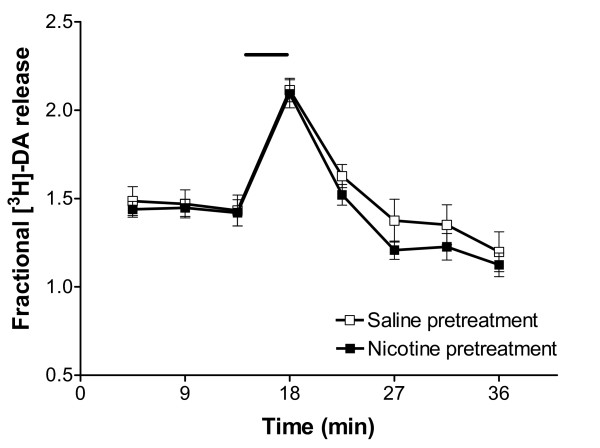
Effect of nicotine pre-treatment on basal and electrically evoked release of [^3^H]-DA from VTA slices. The rats received daily subcutaneous injections of nicotine (0.4 mg kg^-1^, *n *= 6) or saline (*n *= 6) for 14 consecutive days and the experiments were performed 24 hours after the last injection. An electrical stimulation (20 mA, 2 Hz, 4 min, black bar) was applied to the VTA slices 14 min after the beginning of sample collection. The data are expressed as fractional [^3^H]-DA release and represented as mean ± s.e.mean.

The release of [^3^H]-DA from the VTA slices of rats that received a chronic nicotine treatment was compared with the overflow observed in saline-control animals. Neither the basal nor the electrically stimulated release of [^3^H]-DA was significantly affected by the drug pre-treatment (Figure 5).

### Effect of baclofen on the release of [3H]-DA from VTA of nicotine-treated rats

**Figure 6:**
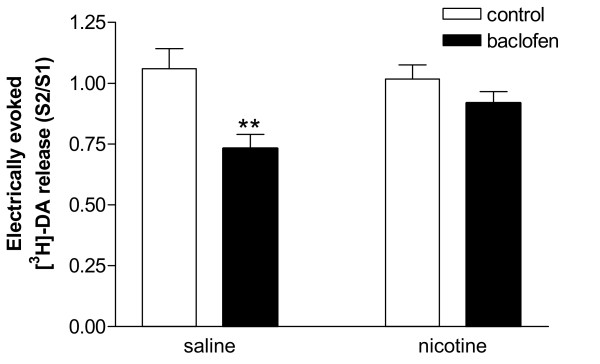
Effect of baclofen on the electrically evoked release of [^3^H]-DA from VTA slices of rats pretreated with nicotine or saline. The rats received daily subcutaneous injections of nicotine (0.4 mg kg^-1^, *n *= 5) or saline (*n *= 5) for 14 consecutive days and the experiments were performed 24 hours after the last injection. Two electrical stimulations (20 mA, 2 Hz, 4 min) were applied to the slices 14 min (S1) and 59 min (S2) after the beginning of sample collection. Baclofen (10 μM) was added to the superfusion buffer 36 min before and during S2. Control tissue was perfused with buffer alone during both S1 and S2. Data are expressed as mean ± s.e.mean and compared by ANOVA for repeated measures. **p < 0.01 vs saline-control (Bonferroni post-test).

The effect of baclofen on the VTA [^3^H]-DA release in rats pre-treated with nicotine was assessed and compared with the effect of the drug in saline-control animals (Figure 6). The addition of baclofen (10 μM) to the superfusion buffer for 36 min before and during S2, significantly reduced the evoked release of [^3^H]-DA from VTA slices of rats pre-treated with saline (S2/S1: control, 1.06 ± 0.08; baclofen, 0.73 ± 0.06; P < 0.01, ANOVA for repeated measures, Bonferroni post-test, *n *= 5 rats). By contrast, in the rats pre-treated with nicotine, the addition of baclofen to the superfusion buffer did not produce any significant effect on the evoked release of [^3^H]-DA (S2/S1: control, 1.02 ± 0.06; baclofen, 0.92 ± 0.05) (Figure 6).

## Discussion

In the present study we have characterised the effect of baclofen on the release of [^3^H]-DA from ventral tegmental area slices of naïve rats, and used this model for studying the functional status of local GABA_B _receptors after chronic exposure to nicotine.

The electrically induced [^3^H]-DA release from VTA somatodendrites was calcium-dependent and tetrodotoxin-sensitive. This suggests that the release is tightly coupled with voltage-sensitive calcium influx and that it depends on the propagation of action potentials by voltage-dependent sodium channels. Our findings are consistent with the calcium sensitivity and the partial block by tetrodotoxin of the release of endogenous dopamine observed in DA cell body areas using microdialysis [10,40]. They are also consistent with the calcium and tetrodotoxin dependency of the electrically evoked [^3^H]-DA release from slices of the ventral tegmentum [41]. Therefore, under the experimental conditions used in the present study, release of preloaded [^3^H]-dopamine appears to be of neuronal origin and to have physiological relevance.

Although the radioactivity measured in the collected effluent may consist of a mixture of neurotransmitters and metabolites, the amount of tritium released from rat brain slices after electrical stimulation has been previously shown to represent a close estimation of the release of labelled or endogenous DA release [42,43]. Furthermore, the release of metabolites during the superfusion was inhibited by the presence of the monoamine oxidase inhibitor pargyline in the superfusion buffer [44]. Therefore, under our experimental conditions, the overflow of tritium from VTA slices seems likely to represent, predominantly, [^3^H]-DA and closely resembles exocytotic release of DA, as it was dependent on the presence of calcium in the superfusion buffer.

The VTA receives GABAergic input from both interneurones and descending projections from the basal forebrain, innervating GABA_A _and GABA_B _receptors, respectively [4,45]. Activation of GABA_B _receptors, located both postsynaptically on dopaminergic neurones and presynaptically on glutamatergic nerve terminals [29], decreases the spontaneous pacemaker-like activity and the burst firing of VTA DA cells [4,31]. In addition, baclofen microinjected into the VTA has been shown to decrease somatodendritic release of dopamine in this midbrain region as monitored by *in vivo *microdialysis [32,33]. The activation of GABA_B _receptors has also been reported to inhibit the potassium- or electrically evoked release of dopamine from various regions of the mammalian brain *in vitro *[46,47]. However, to date, there is a lack of information regarding the effect of GABA_B _receptor activation on the release of somatodendritic dopamine from the isolated VTA. Therefore, in the present study we have demonstrated that baclofen dose-dependently reduces the electrically evoked release of [^3^H]-DA from ventral tegmental area slices of naïve rats. This effect appears to be mediated by activation of GABA_B _receptors, since it was abolished by superfusion of the tissue with the selective receptor antagonist CGP55845A [48].

The existence of a tonic GABA_B _receptor-mediated inhibition of somatodendritic DA release has been suggested by the evidence that, *in vivo*, the administration of CGP55845A into the ventral tegmental area produces a dose-dependent increase in VTA dopamine levels [49]. By contrast, our results demonstrate that the GABA_B _receptor antagonist CGP55845A does not have any significant effect on the release of [^3^H]-DA when applied on its own to the VTA slices. The apparent discrepancy between the two studies may be ascribed to the fact that we have used isolated tissue, which is deprived of the tonic GABA input arising from the forebrain, whereas in the work of Giorgetti et al. [49] the long-loop projections to the VTA are indeed intact. In fact, the innervation of VTA GABA_B _receptors originates primarily from projection neurones located in the nucleus accumbens and the ventral pallidum, while the GABA interneurones appear to innervate mainly GABA_A _receptors [4,50].

Interestingly, previous neurochemical studies have shown that activation of GABA_B _receptors in the ventral tegmental area of naïve rats inhibits the release of dopamine not only in the cell body area, but also in mesocorticolimbic terminal regions, such as the nucleus accumbens [33] and the prefrontal cortex [15]. The reduction of mesocorticolimbic dopamine release might represent a likely mechanism by which baclofen attenuates nicotine self-administration in rats when microinjected into the ventral tegmental area [34]. However, to date, this hypothesis has not been confirmed and little information is known about the neurochemical mechanisms underlying the GABA_B _receptor-mediated modulation of nicotine reinforcement, as well as about the pharmacological interaction between nicotine and GABA_B _receptors. With the present study we demonstrate that while baclofen significantly reduces the electrically induced release of [^3^H]-DA from the VTA of saline-control rats, it has no effect on the evoked monoamine release from VTA slices of nicotine pre-treated rats. This finding represents the first demonstration that chronic exposure to nicotine might result in reduced GABA_B_-mediated inhibition of VTA dopaminergic neurones. This hypothesis is also consistent with preliminary *in vivo *studies performed in our laboratory showing that, after chronic pre-treatment of the rats with nicotine, microinfusions of baclofen into the VTA failed to reduce both the spontaneous and the nicotine-evoked overflow of dopamine in this midbrain region (D. Amantea, unpublished observation). Taken together, our findings suggest that after chronic nicotine the GABA_B _receptor may be desensitised, and this would result in a reduced inhibitory control of VTA dopaminergic cells, thereby facilitating a more sustained increase in the responses of mesolimbic neurones to nicotine. Interestingly, the inhibitory action of baclofen on VTA DA cells may be accounted for by stimulation of GABA_B _receptors located on dopaminergic and/or glutamatergic neurones [28,29]. This suggests that the effect observed after chronic administration of nicotine may be ascribed to a reduced sensitivity of GABA_B _receptors located either on DA cell bodies or, presynaptically, on glutamatergic terminals impinging onto DA neurones. The latter hypothesis would lead to the speculation that a chronic treatment with nicotine might result in increased excitatory input to the VTA, due to a reduced GABA_B_-mediated inhibitory control. Therefore, disinhibition or increased excitatory input to midbrain DA cells might contribute to the augmented dopamine output observed in the nucleus accumbens after a challenge injection of nicotine in rats pretreated with the drug [36,37]. Desensitisation of GABA_B _receptors located in the VTA has also been reported to occur after chronic cocaine administration, as the drug treatment resulted in reduced functional coupling of the receptor to G-proteins [51]. However, we have previously demonstrated that GABA_B _receptor expression and coupling to G-proteins in the ventral tegmental area of the rat are not altered after chronic exposure to nicotine [52], suggesting that desensitisation might occur at other levels, perhaps on downstream effector mechanisms. Nevertheless, it cannot be ignored that the use of *in vitro *autoradiography did not allow us to discriminate between receptor subpopulations, including receptor subtypes involved in different functions. Thus, if the GABA_B _receptors directly implicated in the control of dopamine release from the VTA represent only a small fraction of the overall receptor population in this area, autoradiographic analysis would not be sufficiently sensitive to evaluate receptor modifications occurring after chronic exposure to nicotine. Further studies aimed at characterising the mechanisms involved in GABA_B _receptor desensitisation following chronic nicotine treatment are required to clarify this issue.

Not surprisingly, basal and evoked release of dopamine from VTA slices of rats chronically injected with nicotine did not differ from release obtained from saline-control tissue. This confirms that sensitised dopaminergic responses to the drug depend on the activation of nicotinic receptors (nAChRs) and are not the result of a generic increase in neuronal activity. Similar results have been obtained in striatal synaptosomes from rats pretreated with the nAChR agonist anatoxin-a for 7 days: while the drug pre-exposure increased the nicotine-stimulated release of [^3^H]-DA from the *in vitro *preparation, no difference was found in the K^+^-evoked release between the drug pretreated animals and the saline-injected controls [53]. Thus, although the electrical stimulation used in the present study does not provide information about desensitisation or up-regulation of nAChRs, it nevertheless served as a reliable model to study the functional status of the GABA_B _receptor in the ventral tegmental area of rats pretreated with nicotine.

## Conclusions

In conclusion, we have demonstrated that activation of GABA_B _receptors inhibits the release of preloaded [^3^H]-DA from somatodendritic fields of VTA neurones in naïve rats. This effect appears to be reduced in rats chronically treated with nicotine, suggesting that subsensitivity of GABA_B _receptors in the VTA might occur as a result of the drug treatment. This, in turn, would lead to disinhibition of VTA dopaminergic cells, which might contribute to the increased activity of mesocorticolimbic neurones following repeated exposure to nicotine.

## Methods

### Drugs

[^3^H]-Dopamine (specific activity 47 Ci/mmol) was obtained from Amersham (Buckinghamshire, UK). (-)-Baclofen (CGP11973A) and CGP55845A were generous gifts from Novartis Pharma (Basel, Switzerland). (-)-Nicotine hydrogen tartrate salt, nomifensine, pargyline, tetrodotoxin and ethylene glycol-bis (b-amino ethyl ether) tetraacetic acid (EGTA) were purchased from Sigma-Aldrich (Dorset, UK). All the other chemicals used in this study were obtained from Fisher Scientific (Leicestershire, UK).

### Subjects

Male Wistar rats (weight 250–280 g) were maintained on a 12-h light/dark schedule (on 6:00–18:00), with free access to food and water.

For the drug treatments, rats, initially weighing 150–180 g, received subcutaneous injections of nicotine (0.4 mg kg^-1^, expressed as free-base) or vehicle (0.9% NaCl, 1 ml kg^-1^) for 14 consecutive days, once a day, between 9:30 and 10:30 a.m. Experiments were performed 24 hours after the last injection. All procedures were carried out in accordance to the UK Animals (Scientific Procedures) Act, 1986.

### Tissue preparation

Animals were sacrificed by stunning followed by decapitation. The brains were rapidly removed from the skull and cooled for 2–3 min in ice-cold superfusion buffer of the following composition (in mM): NaCl, 118; KCl, 4.7; CaCl_2_, 1.3; MgCl_2_, 1.2; NaH_2_PO_4_, 1; NaHCO_3_, 25; glucose, 11.1; Na_2_EDTA, 0.004 and ascorbic acid, 0.3 (pH 7.4), saturated with 95% O_2_/5% CO_2_.

**Figure 1:**
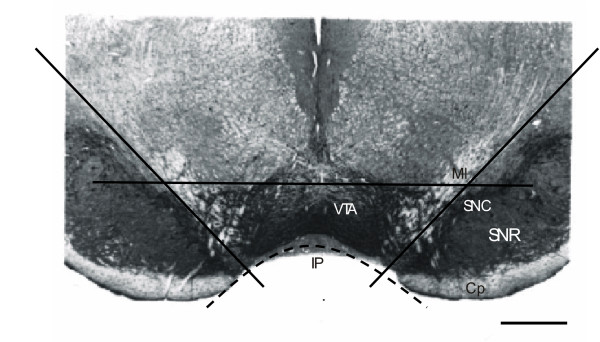
Photomicrograph of a representative coronal section of rat brain labelled with a polyclonal antibody directed against tyrosine hydroxylase. The picture shows the orientation of the cuts (dark lines) for the dissection of the ventral tegmental area (VTA). The basal cerebral peduncle (Cp) was used as a visual landmark to obtain the coronal section containing the VTA. To remove the substantia nigra (pars compacta, SNC, and reticulata, SNR) from this slice, two transversal cuts were made along the medial lemniscus (Ml) on either side. Finally, the VTA was dissected out by making a horizontal cut 1.5 mm dorsal to the ventral edge of the section and stripped of the interpeduncular nucleus (IP) by cutting 0.5 mm above the same edge. Scale bar is 1 mm.

Each brain was dissected according to visual anatomical landmarks and the atlas of Paxinos & Watson [54]. To obtain the coronal section containing the VTA, the brain was placed ventral side up and two parallel cuts were made at the level of the mammillary body and the medial interpeduncular nucleus (approximately 5–6 mm posterior to bregma), using as landmark the basal cerebral peduncle. Two transversal cuts were made in correspondence of the medial lemniscus, to remove the substantia nigra from the slice. Finally the ventral part of the section was dissected out by making a horizontal cut 1.5 mm dorsal to the ventral edge of the section and deprived of the interpeduncular and mammillary nuclei by cutting 0.5 mm above the same edge. The procedure used for the dissection of the VTA is illustrated in Figure 1, which shows a photomicrogaph of a representative coronal slice immunohistochemically stained for tyrosine hydroxylase.

The dissected VTA was cross-chopped (250 μm × 250 μm) with a McIlwain tissue chopper and the tissue slices were washed and resuspended in ice-cold superfusion buffer.

### Tissue superfusion

The VTA slices were pre-incubated in oxygenated superfusion medium at 37°C for 15 min, followed by incubation with 0.1 μM [^3^H]-DA for 30 min, in the dark and in the presence of 10 μM pargyline. The incubation was terminated by washing the slices three times with buffer containing 2.5 μM nomifensine.

150 μl aliquots of slice suspension (1.5 to 2.0 mg of tissue) were transferred to each chamber of a Brandel 2000 superfusion apparatus. The tissue was superfused at a rate of 0.5 ml min^-1 ^with oxygenated buffer maintained at 35°C and containing 2.5 μM nomifensine, to block dopamine uptake, and 10 μM pargyline to ensure that [^3^H] overflow represented primarily [^3^H]-DA rather than its metabolites [44].

After 36-min pre-superfusion, the effluent was collected in consecutive fractions of 4 min 30 sec each. The release of [^3^H]-DA was induced by electrical field stimulation (20 mA, 2 Hz for 4 min) using a Brandel constant current stimulator. Two stimulations occurred 14 min (S1) and 59 min (S2) after the beginning of sample collection.

Test drugs were added to the superfusion medium 36 min before the second stimulation. To determine the effects of calcium and tetrodotoxin (TTX), slices were perfused with Ca^2+^-free buffer (in the presence of 1 mM EGTA), or buffer containing 1 μM TTX, for 15 min before and during S1. The superfusion medium was then replaced with normal buffer until the end of superfusion and during the second stimulation.

### Liquid scintillation counting

At the end of the superfusion, the slices with their filters were removed from the chamber, suspended in 1 ml of buffer, and sonicated. OptiPhase 'HiSafe' 3 scintillation fluid (Perkin Elmer, UK) was added to each vial and the radioactivity content in the superfusion samples and in the tissue slices was assayed by liquid scintillation counting using a Tri-Carb^® ^1500 liquid scintillation analyser (Packard Bioscience Company), programmed to count for tritium, 3 minutes per vial, at an efficiency of 60%. The number of disintegrations per minute (d.p.m.) was measured in order to determine the concentration of tritium in each sample.

### Data analysis

For each time point (4 min 30 sec), release of radioactivity was expressed as fractional release, *i.e.*, as a percentage of the amount of radioactivity in the tissue at the beginning of that collection. The electrically evoked release was expressed as the mean of the increased fractional release above baseline in the two fractions after the beginning of stimulations. Basal release, in turn, was calculated as the mean of the amount of radioactivity present in the three samples just before each period of electrical stimulation.

Results were expressed as mean ± s.e.mean of *n *independent experiments conducted in either triplicate or quadruplicate. For statistical analysis one-way ANOVA with Dunnett's post hoc test was used to compare values of S2/S1 in the presence of a drug versus values of S2/S1 from control slices superfused with buffer alone. When nicotine or saline were administered to the animals, values of S2/S1 were analysed by ANOVA with repeated measures (with or without baclofen) with pre-treatment as factor analysed, and post hoc comparisons were made using the Bonferroni test. The accepted level of significance was *p *< 0.05.

### Histology

Immunohistochemistry was performed on naïve rat brain during preliminary experiments aimed at characterising the exact orientation of the cutting for the dissection of the VTA (Figure 1). Animals were sacrificed by stunning and decapitation and their brains rapidly removed from the skull. A coronal section containing the VTA was obtained as described above and the tissue was post-fixed in 4% paraformaldehyde (BDH) for 48 hours at 4°C. Serial coronal sections (30 μm thick) were cut using a vibratome and washed in 0.01 M phosphate buffered saline (PBS, Sigma-Aldrich), pH 7.4. Slices were incubated with 3% hydrogen peroxide (H_2_O_2_) for 30 min, followed by incubation with 0.2% Triton X-100 (Sigma-Aldrich) for 20 min at room temperature.

After a 1-hour pre-incubation in 10% normal goat serum (NGS, Vector), the primary antibody (rabbit polyclonal antibody to tyrosine-hydroxylase, Affiniti) was applied at a final concentration of 1:1000 (in 0.01 M PBS) and the sections were allowed to incubate overnight at 4°C. After three washes with fresh buffer, the slices were incubated for 90 minutes at room temperature with a biotinylated secondary goat anti-rabbit antibody (1:200 dilution, Chemicon).

Immunoreactivity was visualised by the avidin-biotin complex method of detection (Vectastain Elite ABC Kit, Vector) using 3,3'diaminobenzidine (DAB, peroxidase substrate kit, Vector) as the chromogen.

## Authors' contributions

DA carried out the experiments, participated in the design of the study and drafted the manuscript.

NGB conceived the study, and participated in its design and coordination.

All authors read and approved the final manuscript.
